# Angiotensin-converting enzyme 2 inhibits lung injury induced by respiratory syncytial virus

**DOI:** 10.1038/srep19840

**Published:** 2016-01-27

**Authors:** Hongjing Gu, Zhengde Xie, Tieling Li, Shaogeng Zhang, Chengcai Lai, Ping Zhu, Keyu Wang, Lina Han, Yueqiang Duan, Zhongpeng Zhao, Xiaolan Yang, Li Xing, Peirui Zhang, Zhouhai Wang, Ruisheng Li, Jane J. Yu, Xiliang Wang, Penghui Yang

**Affiliations:** 1 State Key Laboratory of Pathogens and Biosecurity, Beijing Institute of Microbiology and Epidemiology, Beijing 100071, China; 2 Key Laboratory of Major Diseases in Children and National Key Discipline of Pediatrics, Capital Medical University; Beijing Pediatric Research Institute, Beijing Children’s Hospital, Capital Medical University, Beijing, 100045, China; 3 Chinese PLA General Hospital, 1000853, China; 4 Beijing302 Hospital, Beijing, 100039, China; 5 Division of Pulmonary, Critical Care and Sleep Medicine, Department of Internal Medicine, College of Medicine, University of Cincinnati, 231 Albert Sabin Way, CVC4926, Cincinnati, OH 45267, USA

## Abstract

Respiratory syncytial virus (RSV) infection is a major cause of severe lower respiratory illness in infants and young children, but the underlying mechanisms responsible for viral pathogenesis have not been fully elucidated. To date, no drugs or vaccines have been employed to improve clinical outcomes for RSV-infected patients. In this paper, we report that angiotensin-converting enzyme-2 (ACE2) protected against severe lung injury induced by RSV infection in an experimental mouse model and in pediatric patients. Moreover, ACE2 deficiency aggravated RSV-associated disease pathogenesis, mainly by its action on the angiotensin II type 1 receptor (AT1R). Furthermore, administration of a recombinant ACE2 protein alleviated the severity of RSV-induced lung injury. These findings demonstrate that ACE2 plays a critical role in preventing RSV-induced lung injury, and suggest that ACE2 is a promising potential therapeutic target in the management of RSV-induced lung disease.

Human respiratory syncytial virus (RSV) is a non-segmented, negative-sense, single-stranded RNA virus belonging to the *Paramyxoviridae* family. RSV is the most common virus responsible for acute and severe lower airway diseases in infants and young children, with nearly all children experiencing at least one RSV infection by the age of 2 years^1^. According to the World Health Organization (WHO), approximately 34 million new pediatric cases of RSV-associated lower airway disease occur annually worldwide; most cases occur in developing countries, with a mortality rate of nearly 200,000 annually^2^,^3^.

Despite the significant impact of RSV infection on infant morbidity and mortality, treatment options remain largely limited to supportive care^4^. Currently, Ribavirin is the only antiviral drug that has been approved for treatment of RSV-infected airway diseases, although issues with its efficacy and side effects restrict its application in immunocompromised patients^5^. The high incidence of RSV infection and its potential to cause severe respiratory tract disease in vulnerable young children, combined with the limited availability of therapeutic options, highlight an urgent unmet global human health need.

The renin-angiotensin system (RAS) plays a major role in maintaining blood pressure, and electrolyte and fluid homeostasis^6^,^7^,^8^. In 2000, ACE2, a homolog of the angiotensin-converting enzyme (ACE) gene was reported to negatively regulate RAS by converting angiotensin (Ang)-II to Ang-1–7^9^. Previous studies, from our group and others, have demonstrated that ACE2 protects against severe acute lung injury (ALI) that can be triggered by sepsis, acid aspiration, severe acute respiratory syndrome (SARS), and the lethal avian influenza A H5N1 virus^10^,^11^,^12^. Moreover, ACE2 has been identified as the receptor of the SARS coronavirus^11^, and has also been found to modulate innate immunity and influence the composition of gut microbiota^13^. More importantly, a recombinant ACE2 protein has proven to be effective in improving lung pathologies associated with ALI and acute respiratory distress syndrome (ARDS) induced by viruses including SARS and influenza^12^,^14^,^15^. Currently, a soluble version of recombinant ACE2 is being tested in phase II clinical trials for patients with ALI^14^.

The mechanisms underlying RSV-induced lung disease and the associated long-term consequences remain incompletely understood, although lung inflammatory responses likely play a central role in promoting pathogenesis^16^,^17^. RSV infection can progress to ARDS, the most severe form of ALI that further contributes to morbidity^18^ in infants^19^,^20^,^21^. Notably, our recent study showed that ACE2 is involved in influenza H7N9-induced lung injury and that use of a recombinant ACE2 protein alleviates the severity of ALI^15^. In this study, we hypothesized that the RAS system mediates the severity of RSV-induced lung injury. Our findings demonstrate that ACE2 plays an important role in RSV-induced lung injury, and that administration of a recombinant ACE2 protein attenuates the severity of lung injury in a preclinical model of RSV infection.

## Methods

### Animals

Three-week-old wild-type (WT) C57BL/6 (abbreviated to B6) mice (Experimental Animal Center, Beijing, China), and three-week-old ACE2 knockout (abbreviated to KO) mice (B6 background) were housed in the animal facility at Beijing Institute of Microbiology and Epidemiology in accordance with institutional guidelines. All experiments involving human subjects were performed in accordance with the guidelines and regulations of Beijing Institute of Microbiology and Epidemiology.

All experimental protocols were approved by the Institutional Animal Care and Use Committee of Beijing Institute of Microbiology and Epidemiology (ID: SYXK2010-005) and carried out in accordance with the approved guidelines.

### Experimental mouse models

The wild-type RSV virus (abbreviated to BJ016) used in this study was isolated from a confirmed RSV-infected patient in Beijing Children’s Hospital. The genomic sequence of BJ016 is available in the GenBank database (accession number KC978856). BJ016 and A2 virus were amplified in Hep-2 cells. After freeze-thaw, the infected cells were harvested by centrifugation at 1,600 × g for 20 min at 4 °C; the supernatants were concentrated by centrifugation at 36,000 × g (Beckman rotor SW401) and the concentrate was re-suspended in 1X PBS. Viral suspensions were overlaid onto a discontinuous sucrose gradient by centrifugation at 42,000 × g; the purified BJ016 or A2 virus bands were collected, diluted with PBS, and concentrated by centrifugation at 30,000 rpm for 1 h. Purified viruses were re-suspended in 1X PBS and stored at −70 °C.

For RSV-induced lung injury, 3-week-old WT B6 mice were anesthetized with 20 μl 1% (w/v) pentobarbital sodium, and then inoculated intranasally (IN) with 2 × 10^6.5^ PFU of BJ016 or 20 μl 1X PBS (mock-infection). For all mice, body weight was recorded daily before and after viral infection. Progression-free survival was evaluated at the time of viral infection. For UV-inactivation, BJ016 viral suspensions at 0.8 ml/well in a six-well plate were exposed to ultraviolet (UV) (approximately 5 inches from UV light source) for 60 min in a biosafety cabinet.

### Treatment with recombinant ACE2 protein and AT1R/AT2R inhibitors

Drug treatment via intravenous (IV) injection was initiated 1 day before RSV infection, at the following dosages: recombinant human ACE2 protein (R&D Systems, Minneapolis, MN, USA; Sino Biological Inc., North Wales, PA, USA), 0.1 mg/kg; losartan, an AT1R inhibitor (Merck, Kenilworth, NJ, USA), 15 mg/kg; PD123.319, an AT2R inhibitor (Tocris Bioscience, Bristol, UK), 15 mg/kg; or 1X PBS (vehicle control).

### Measurement of Angiotensin II levels

Ang II levels were measured as described elsewhere^22^.

### Western blotting

Lung tissues were lysed in RIPA buffer. The following antibodies for Western blotting were used: rat polyclonal anti-ACE2 antibodies and rabbit monoclonal anti-ACE antibodies (all R&D Systems).

### Histological examination

Following anesthetization with pentobarbital sodium, 3-week-old mice were infected IN with 2 × 10^6.5^ PFU BJ016, and sacrificed on day 5 (days post infection; DPI). For hematoxylin and eosin (H&E) staining, the lungs from each mouse were fixed in 10% formalin, embedded in paraffin, cut into 4-μm sections, and stained with H&E to analyze pathological changes in lung tissue. The number of inflammatory cells was counted, and data were analyzed statistically and presented as the number of cells per 200× field.

### Lung wet-to-dry weight ratio

Mice were sacrificed at 5 DPI. The lungs were weighed and then dehydrated at 68 °C for 48 h. The severity of pulmonary edema was measured using the lung wet-to-dry weight ratio.

### Viral titration

Virus titers were measured in the supernatants of lung homogenates from mice at 5 DPI as described previously^23^. Briefly, Hep-2 cells were cultured as monolayers in 24-well plates, and infected with serial 10-fold dilutions of lung suspension in DMED/F12 (1:1). The overlay was prepared with agar (Sigma-Aldrich, St Louis, MO, USA) at a final concentration of 1% DMEM/F12=1:1 (Hyclone Laboratories, South Logan, UT, USA) supplemented with 1% fetal calf serum (Gibco, Waltham, MA, USA). Hep-2 cells were cultured for 7 days at 37 °C and 5% CO_2_. Cells were stained with crystal violet, and viral plaques were counted. Viral titers were calculated using the Reed and Muench method^24^, and expressed as Log_10_ PFU/g of lung tissue.

### Patient recruitment

RSV-infected patients and healthy children were recruited from Beijing Children’s Hospital, the China PLA General Hospital, and Beijing 302 Hospital. Subjects provided informed consent for participation in this study.

### Determination of plasma angiotensin II

Levels of angiotensin II (Ang II) were determined using ELISA according to the manufacturer’s instructions (RapidBio Lab, Calabasas, CA, USA). Plasma samples were analyzed in triplicate.

### Statistical analysis

All data are presented as means ± SEM. Measurements at single time-points were analyzed using ANOVA, and survival data were analyzed using Kaplan-Meier survival analysis. All statistical analyses were performed using GraphPad Prism software (ver. 5.0; San Diego, CA, USA). A value of *p* < 0.05 was considered statistically significant. All experiments were performed in triplicate at least.

## Results

### RAS is involved in the process of RSV virus infection

**Figure 1:**
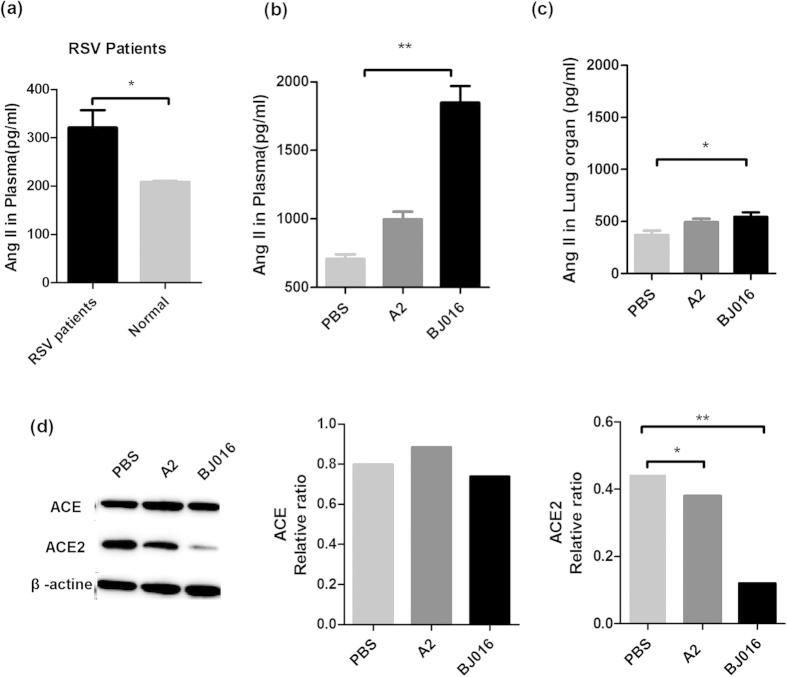
Angiotensin-converting enzyme-2 (ACE2) plays a critical role in respiratory syncytial virus (RSV)-induced lung injury. (**a**) Plasma angiotensin II (Ang II) levels in healthy children and RSV-infected patients were measured using enzyme immunoassays. (**b**) Plasma Ang II levels in control, wild-type (WT) RSV virus (RSV BJ016) and A2 virus-infected mice on day 3 (*n* = 8 per group). (**c**) Ang II levels in the lungs of control, RSV BJ016 and A2 virus-infected mice on day 3 were examined using enzyme immunoassays (*n* = 8 per group). (**d**) ACE2 expression in lung homogenates of control, RSV BJ016 and A2 virus-infected mice were detected by Western blotting. Mice were sacrificed on day 3 post infection.*p < 0.05; **p < 0.01 (two-tailed t-test).

To determine the role of ACE2 in RSV-induced lung injury, we first measured the levels of Ang II in the plasma of RSV-infected patients. In total, 34 pediatric patients and 20 healthy children were recruited from Beijing Children’s Hospital, the China PLA General Hospital, and Beijing 302 Hospital in 2013 and 2014. The clinical characteristics underlying the conditions and outcomes of these individuals are described in Tables S1 and S2. All 34 patients were confirmed to carry solely the RSV viral genomic fragment using PCR analysis. Concomitantly, plasma levels of Ang II were significantly elevated in RSV-infected patients relative to healthy subjects (p < 0.05; Fig. 1a). To determine any correlation between plasma levels of Ang II and the onset of RSV infection, we collected plasma samples from these individuals at early- and late-stages of infection. Plasma levels of Ang II were higher in the early-stage (up until 8 days from onset) than in the late-stage (after 10 days from onset), indicating that plasma levels of Ang II decreased rapidly from the infection to the recovery phase of RSV infection (Figure S1a). Taken together, our data indicate that the renin-angiotensin system plays an important role in the host response against RSV infection.

To evaluate the direct effect of RSV infection on RAS further, mice were infected IN with live RSV BJ016 or A2 virus. Three days after viral infection, plasma levels of Ang II were measured. RSV A2 viral infection resulted in a significant increase in Ang II levels relative to mock infection (*p* < 0.05; Fig. 1b). Interestingly, RSV BJ016 infection led to a greater increase in Ang II levels than did infection with A2 virus (*p* < 0.01; Fig. 1b). Concomitantly, Ang II levels in lung homogenates were moderately elevated in mice infected with RSV BJ016 (*p* < 0.05) or A2 virus relative to mock infection (Fig. 1c). To determine the mechanism responsible for the increased levels of Ang II, we examined the protein levels of ACE2, which converts Ang II to Ang 1–7. Accumulation of ACE2 protein was reduced in mice infected with RSV BJ016 or A2 virus, relative to mock infection (Fig. 1d). Importantly, ACE2 protein levels were dramatically decreased in lung homogenates from mice infected with RSV BJ016 virus at 3 days post-infection (*p* < 0.01; Fig. 1d). Interestingly, levels of ACE protein were comparable in the lungs of mice infected with RSV or mock virus (Fig. 1d). These data demonstrate that RSV infection leads to a decrease in ACE2 expression and an increase in Ang II levels in preclinical models, further supporting the critical role of RAS in RSV infection.

### ACE2 deficiency increases the severity of RSV-induced lung injury

**Figure 2:**
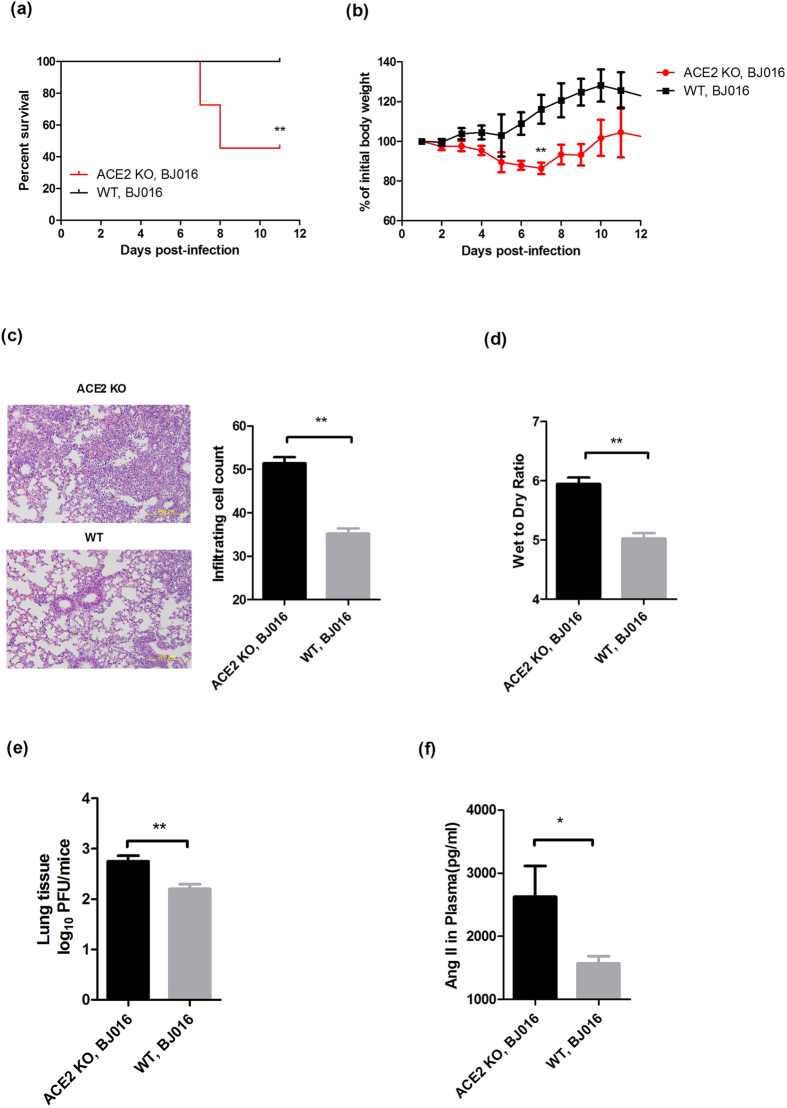
Loss of ACE2 increases severity of RSV BJ016 virus-induced lung injury. (**a**) Survival rates of WT and ACE2 knockout (KO) mice (*n* = 10 per group). (**b**) Body weight changes of WT and ACE2 KO mice (*n* = 10 per group). (**c**) Hematoxylin and eosin (H&E) staining and infiltrating cell counts (*n* = 200 fields) in lung sections of WT C57BL/6 (B6) and ACE2 KO mice (*n* = 5) at 5 days post injection (DPI). (**d**) Lung wet-to-dry weight ratios from WT B6 and ACE2 KO mice (n = 8) at 5 DPI. (**e**) Lung viral titers in RSV BJ016 virus-infected WT B6 and ACE2 KO mice (*n* = 8) at 5 DPI. (**f**) Detection of plasma Ang II in WT B6 and ACE2 KO mice (*n* = 8) at 5 DPI.

To investigate the impact of ACE2 on live RSV-induced clinical signs associated with lung injury, we infected ACE2 knockout (ACE2 KO) or wild-type (WT) mice with RSV BJ016 virus, and measured overall survival. Fourteen days post infection, 100% of WT mice survived, whereas only 40% of ACE2 KO mice survived (Fig. 2a). Seven days post RSV infection, the average body weight of ACE2 KO mice decreased by 77% relative to that of WT mice (*p* < 0.01; Fig. 2a). Meanwhile, the lung histopathology scores, as defined by leukocyte infiltration cell counts, were significantly reduced in ACE2 KO mice compared with WT mice (Fig. 2c). Moreover, lung edema, defined by the lung wet-to-dry weight ratio, was significantly greater in RSV-infected ACE2 KO mice compared with WT mice (*p* < 0.01; Fig. 2d), indicating a more severe lung injury phenotype. Consistent with these findings, viral titers in lung tissue of RSV-infected ACE2 KO mice were significantly higher by approximately five-fold relative to those of RSV-infected WT mice (*p* < 0.01; Fig. 2e). Furthermore, plasma levels of Ang II were significantly elevated in ACE2 KO mice compared with WT mice (*p* < 0.05) at 3 days post BJ016 infection (Fig. 2f). Similar findings were observed in ACE2 KO and WT mice infected with RSV A2 virus. In an independent experiment, 100% of WT mice were alive at 14 DPI, whereas only 60% of ACE2 KO mice survived (Figure S2a). Concomitantly, body weight decreased in ACE2 KO mice compared with WT mice at 7 DPI (Figure S2b). The lung wet-to-dry weight ratio was significantly higher in RSV A2-infected ACE2 KO mice than in RSV A2-infected WT mice (*p* < 0.01; Figure S2c). Viral titers of RSV A2 virus-infected KO mice were also significantly higher by nearly five-fold compared with WT mice (*p* < 0.01; Figure S2d). Collectively, these results suggest that ACE2 plays a critical role in RSV-induced lung injury. Thus, interfering with ACE2 expression may attenuate disease severity following respiratory RSV infection.

### Recombinant hACE2 reduces the severity of RSV-induced lung injury

**Figure 3:**
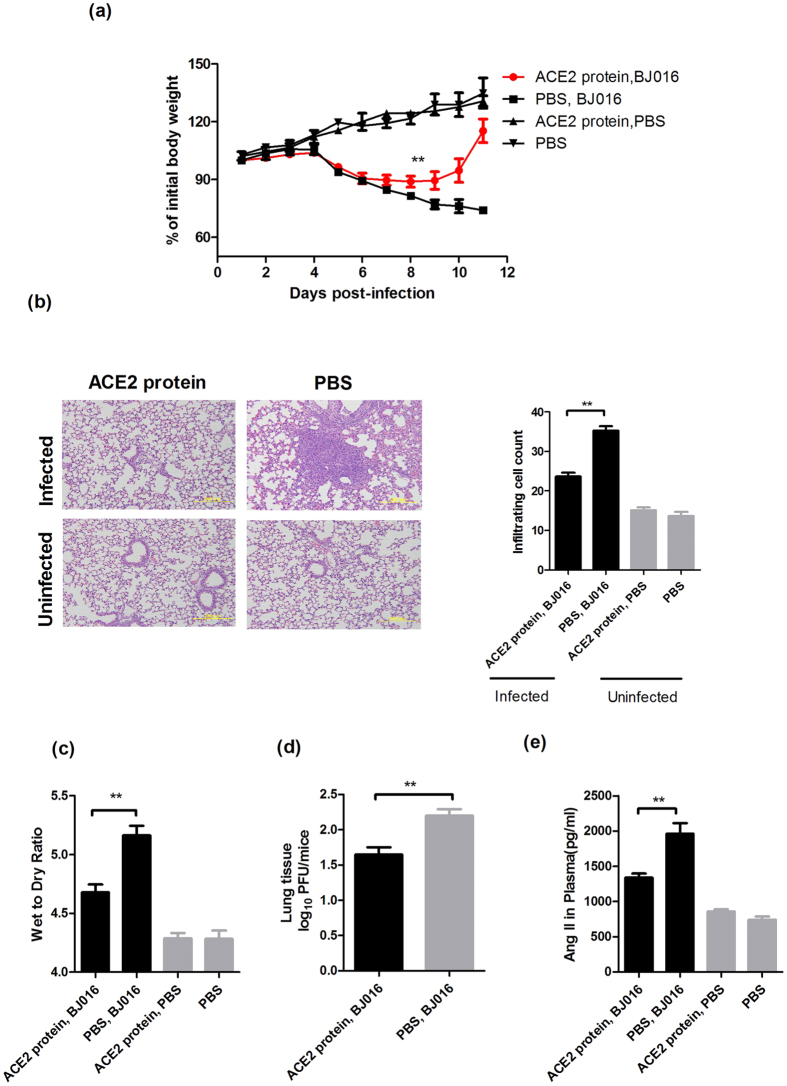
Recombinant hACE2 protects against lung injury induced by RSV BJ016 viral infection in mouse models. (**a**) Body weight changes of RSV BJ016 virus-infected WT mice. Mice were treated intravenously with 0.1 mg/kg soluble recombinant hACE2 protein or control, 1 day before infection, 1 DPI and 3 DPI (n = 10/treatment group). (**b**) H&E staining and infiltrating cell counts (n = 200 fields) in lung sections of RSV BJ016 virus-infected mice at 5 DPI (n = 3). (**c**) Lung wet-to-dry weight ratios in mice infected with RSV BJ016 virus at 5 DPI (n = 5). (**d**) Lung viral titers in mice infected with RSV BJ016 virus at 5 DPI (n = 5). (**e**) Plasma levels of Ang II in RSV BJ016 virus-infected mice treated with hACE2 proteins (n = 6). All experiments were performed in triplicate at least.

To examine whether ACE2 proteins efficiently protect against RSV infection in preclinical models, RSV BJ016 virus-infected WT mice were treated with recombinant hACE2 proteins (0.1 mg/kg)^10^ 1 day before infection, as well as at 1 and 3 DPI. At 8 DPI, a significant increase in body weight was observed -n hACE2-treated mice and vehicle-treated mice (P < 0.01; Fig. 3a). Importantly, hACE2-treated mice exhibited only mild inflammatory reactions, whereas severe histopathological damage, including fragmentation of alveolar walls and infiltration of lymphocytes, was observed in vehicle-treated mice. Concomitantly, leukocyte cell counts were significantly decreased in hACE2-treated mice compared with vehicle-treated mice (p < 0.01; Fig. 3b). Moreover, the lung wet-to-dry weight ratio in hACE2-treated mice was significantly reduced (p < 0.01; Fig. 3c), indicating an attenuation of lung edema mediated by recombinant ACE2 proteins. Additionally, the lung viral titers of hACE2-treated mice were significantly decreased by nearly 30% compared with vehicle-treated mice (*p* < 0.01; Fig. 3d). Furthermore, plasma levels of Ang II were significantly decreased in hACE2-treated mice (*p* < 0.01) at 5 DPI (Fig. 3e). Notably, hACE2-treated mice infected with RSV A2 exhibited mild pathological lung alterations (Figure S2a). The lung wet-to-dry weight ratio in hACE2-treated mice was decreased by 10% compared with vehicle-treated mice (*p* < 0.01; Figure S2b). Viral titers of hACE2-treated mice infected with RSV A2 were also significantly decreased by 30% compared with vehicle-treated mice (*p* < 0.01; Figure S3c). These data therefore demonstrate the therapeutic effects of recombinant hACE2 protein in improving lung physiology and histopathology *in vivo*, and support the potential use of hACE2 protein to alleviate the symptoms of acute lung injury during RSV infection.

### The Ang II receptor AT1R affects the severity of RSV-induced lung injury

**Figure 4:**
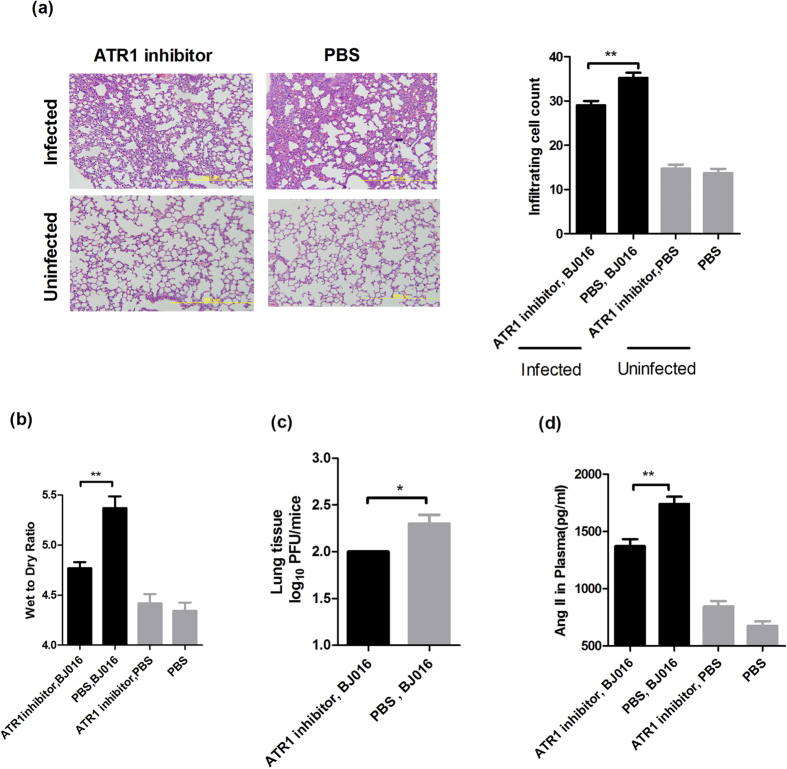
Ang II receptor angiotensin II type 1 receptor (AT1R) regulates RSV BJ016 virus-induced lung injury. (**a**) H&E staining and infiltrating cell counts (*n* = 200 fields) in lung sections of RSV BJ016 virus-infected B6 mice treated with PBS control or AT1R inhibitor (Losartan, 15 mg/kg) at 5DPI. (**b**) Lung wet-to-dry weight ratio of WT mice treated with vehicle control or AT1R inhibitor (losartan, 15 mg/kg) 30 min before RSV BJ016 viral infection (*n* = 8). (**c**) Lung viral titers in WT mice treated with PBS control or AT1R inhibitor (losartan, 15 mg/kg) before RSV BJ016 viral infection (*n* = 8) at 5 DPI. (**d**) Detection of plasma levels of Ang II in WT mice treated with PBS control or AT1R inhibitor (Losartan, 15 mg/kg) 30 min before RSV BJ016 viral infection (*n* = 8), and at 5 DPI (*n* = 8). *p < 0.05; **p < 0.01 (two-tailed t-test).

Because Ang II receptors mediate the action of Ang II, we next assessed the efficacy of inhibitors specific for the AT1 or AT2 receptors (AT1R/AT2R) by examination of *in vivo* clinical signs in the lungs. First, live RSV-infected WT mice that received the vehicle treatment exhibited lung edema and infiltration of inflammatory cells (Fig. 4a). The AT1R inhibitor (Losartan) treatment markedly attenuated the severity of RSV-induced histopathologic alterations in RSV-infected mice (Fig. 4a). Next, the lung wet-to-dry weight ratio was significantly reduced, by 6% (*p* < 0.05), in AT1R inhibitor-treated mice compared with vehicle-treated mice (Fig. 4b). Moreover, lung viral titers decreased markedly in the RSV BJ016 virus-infected WT mice treated with AT1R inhibitor (Fig. 4c). Furthermore, plasma levels of Ang II were also reduced by treatment with the AT1R inhibitor (Fig. 4d). On the contrary, the AT2R inhibitor had no significant effects on the lung wet-to-dry weight ratio (Figure S4a) or viral titers (Figure S4b), suggesting a lack of efficacy of this inhibitor in rescuing the lung injury induced by BJ016 virus-infected WT mice. Therefore, these data support the assertion that both Ang II and AT1R play critical roles in regulating RSV-induced ALI in preclinical models.

### Inhibition of AT1R attenuates RSV-induced lung injury in ACE2 KO mice

**Figure 5:**
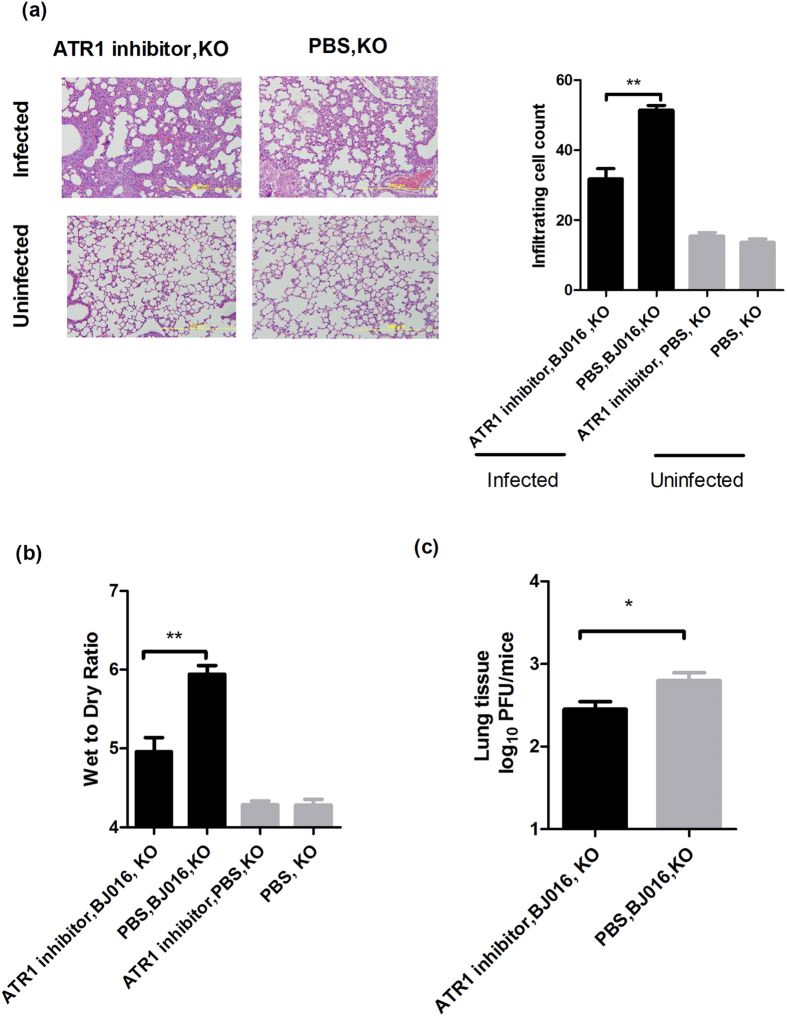
Inhibition of AT1R attenuates RSV BJ016 virus-induced lung injury in ACE2 knockout mice. (**a**) H&E staining and infiltrating cell counts (*n* = 200 fields) in lung sections of RSV BJ016 virus-infected ACE2 KO mice treated with PBS control or AT1R inhibitor (losartan, 15 mg/kg) at 5 DPI. (**b**) Lung wet-to-dry weight ratios of ACE2 KO mice treated with PBS control or AT1R inhibitor (Losartan, 15 mg/kg) 30 min before RSV BJ016 viral infection (*n* = 8). (**c**) Lung viral titers in ACE2 KO mice treated with PBS control or AT1R inhibitor at 5 DPI. *p < 0.05; **p < 0.01 (two-tailed t-test). (**d**) Schematic diagram of the role of the renin-angiotensin system in lung injury and RSV infection.

Our previous demonstration that ACE2 mediates RSV-induced lung injury (Fig. 1), led us to hypothesize that suppression of AT1R attenuates RSV-induced lung injury in ACE2 KO mice. To test this, RSV BJ016 virus-infected ACE2 KO mice were treated with AT1R (15 mg/kg)^10^ at 1 day before infection, 1 DPI and 3 DPI. At 5 DPI, AT1R-treated ACE2 KO mice exhibited mild inflammatory changes, whereas severe histopathological damage was observed in vehicle-treated ACE2 KO mice. Concomitantly, leukocyte cell counts were reduced by 10% in ACE2 KO mice treated with AT1R (*p* < 0.01; Fig. 5a). The lung wet-to-dry weight ratio was decreased by 86% in AT1R-treated ACE2 KO mice compared with vehicle-treated ACE2 KO mice (*p* < 0.01; Fig. 5b). Moreover, lung viral titers were decreased significantly in AT1R-treated ACE2 KO mice compared with vehicle-treated ACE2 KO mice (Fig. 5c). Of note, there was no apparent effect on lung edema (Figure S5a) or viral titers (Figure S5b) in BJ016 virus-infected ACE2 KO mice treated with the AT2R inhibitor, indicating that AT2R was likely not to be involved in the process of RSV-induced lung injury. Based on the above data, we propose that ACE2 may play a crucial role in the process of RSV viral infection, mainly by affecting AT1R (Fig. 5d). Our data suggest that RSV infection causes severe lung injury in an experimental mouse model and in patients, at least in part by modulating the RAS system via down-regulation of the ACE2-AT1R axis.

## Discussion

Previous studies have reported on the use of RSV-induced lung injury models for testing drug efficacy^16^. The selection of a reliable mouse model of RSV infection for investigation of associated clinical abnormalities has been a major challenge in RSV research. The A2 strain was originally isolated from clinical samples in 1961, and has long been the standard laboratory RSV strain used in research^25^,^26^. However, this historical strain induces mild lung pathology in mice compared to other recently isolated strains^27^. Therefore, we attempted to isolate pathogenic viruses from clinical nasopharyngeal swabs collected from pediatric patients, and subsequently identified BJ016. BJ016 virus is an isolated RSV belonging to subgroup A, and causes severe respiratory disease in preclinical models. The hospital-isolated BJ016 strain was used in this study because of its direct clinical relevance. We anticipate that the BJ016 strain will be widely used for the development of animal models for RSV research.

Previous studies have shown that ACE2, expressed in the lungs of patients with pulmonary diseases and also in healthy subjects, is involved in ALI induced by SARS and influenza (lethal H7N9 and H5N1virus)^12^,^15^. Importantly, Yumiko Imai *et al.* revealed that ACE2 is an essential receptor for SARS infection *in vitro* and *in vivo*^11^,^28^, and that it protects individuals from developing severe acute lung failure^11^. Moreover, as ACE2 is a non-specific protease, it would also be interesting to investigate its role in the regulation of cellular metabolites^14^. However, ACE2 is non-functional during lung injury processes induced by various influenza virus subtypes such as H1N1 and H3N2 (data not shown). There are associations between influenza virus infection and ACE2 levels, even with different influenza strains (avian high-path H5N1 versus H7N9)^12^,^15^. Despite the reports that focused on the impact of these viruses on lung pathology, the underlying molecular mechanisms responsible for RAS- and ACE2-mediated- lung injury have not been fully elucidated.

We report here that ACE2 plays an important role in RSV-induced lung injury. This was associated with increased plasma levels of Ang II in RSV-infected patients in China. Nevertheless, a considerably larger cohort of RSV-infected individuals from various regions of China is required to confirm these findings. Live RSV-induced lung injury resulted in significant down-regulation of ACE2 at the early stage after the onset of infection, which controlled the function of RAS, but not in the UV-BJ016 group (Figure S6). We hypothesize that RSV growth is directly or indirectly related to RSV-induced pathology. Moreover, our studies provide a molecular basis for the mechanism of lung injury in patients infected with RSV. Importantly, the levels of Ang II were elevated following down-regulation of ACE2, causing severe lung injury via AT1R during the process of RSV infection (Fig. 5d). Our discovery reveals that common molecular and cellular mechanisms are likely shared by animal models and patients with acid-aspiration^10^, sepsis^29^, SARS-CoV^11^, influenza virus^12^,^15^ or RSV infection.

Moreover, our data demonstrate that ACE2 deficiency aggravates RSV-induced ALI and that administration of a soluble ACE2 recombinant protein ameliorates lung injury *in vivo.* Nevertheless, further studies assessing the potential therapeutic efficacy of recombinant human ACE2 protein are required using different animal models to verify the protective effects of ACE2 against RSV-induced lung injury. Other studies have used cotton rats and monkeys to study RSV pathogenesis and RSV vaccine or drug investigations^30^. The combination of clinical findings and preclinical studies has revealed a critical role of RAS in the pathogenesis of RSV-induced lung injury, and demonstrated that ACE2 plays a key role in the development and progression of RSV infection. Collectively, our findings suggest the intriguing possibility that targeting ACE2 and RAS represents a valuable strategy for treating RSV infections.

Modulation of ACE2 represents a potential novel pharmacological approach to ameliorate RSV-induced lung inflammation. Mechanisms leading to decreased expression/activity of ACE2 have not been fully investigated. In our initial clinical study, we found that elevated plasma concentrations of Ang II correlated with the severity of lung injury. We therefore hypothesize that levels of Ang II in plasma samples of patients with RSV infection may reflect events in the lower respiratory tract. Although our study was performed in a relatively small cohort of RSV-infected subjects, it included a balanced representation of the spectrum of disease that is caused by this pathogen in infancy. Importantly, our preclinical studies suggest that infections caused by other viral respiratory pathogens that are known to cause lung disease in infants may also result in similar inhibition of ACE2 expression. Further clinical studies will be required to clarify this issue. In this regard, we are currently enrolling infants in a prospective study to test patients with a broad spectrum of disease severity caused by RSV. Based on our findings in this study, we suggest that modulation of ACE2 expression and/or blocking lung injury response may represent potential pharmacological approaches to alleviate RSV-induced lung disease.

## Additional Information

**How to cite this article**: Gu, H. *et al.* Angiotensin-converting enzyme 2 inhibits lung injury induced by respiratory syncytial virus. *Sci. Rep.* **6**, 19840; doi: 10.1038/srep19840 (2016).

## Supplementary Material

Supplementary Information
