# Supplementary material for: Angiotensin-converting enzyme 2 inhibits lung injury induced by respiratory syncytial virus
Source: Sci Rep. 2016 Jan 27;6:19840. doi: 10.1038/srep19840 (PMC4728398; doi:10.1038/srep19840)
Supplement: Supplementary Information [file srep19840-s1.doc]

**Supplementary data to:**

**Angiotensin-converting enzyme 2 inhibits lung injury induced by respiratory syncytial virus**

Hongjing Gu^1, *^, Zhengde Xie^2, *^, Tieling Li^3, *^, Shaogeng Zhang^4,*^, Chengcai Lai^1^, Ping Zhu^1^, Keyu Wang^1^, Lina Han^3^, Yueqiang Duan^1^, Zhongpeng Zhao^1^, Xiaolan Yang^1^, Li Xing^1^, Peirui Zhang^4^,Zhouhai Wang^4^, Ruisheng Li^4^, Jane J. Yu^5^, Xiliang Wang^1^, Penghui Yang^1,4^

^1^ State Key Laboratory of Pathogens and Biosecurity, Beijing Institute of Microbiology and Epidemiology, Beijing 100071, China;

^2^ Key Laboratory of Major Diseases in Children and National Key Discipline of Pediatrics (Capital Medical University), Beijing Pediatric Research Institute, Beijing Children’s Hospital, Capital Medical University,, Beijing, 100045, China;

^3^ Chinese PLA General Hospital, 1000853, China;

^4^Beijing302 Hospital, Beijing, 100039, China;

^5^ Division of Pulmonary, Critical Care and Sleep Medicine, Department of Internal Medicine, College of Medicine, University of Cincinnati, 231 Albert Sabin Way, CVC4926, Cincinnati, OH 45267, USA.

^*^ All these authors contributed equally to this work.

Correspondence and requests for materials should be addressed to X.W ([xiliangw@126.com](mailto:xiliangw@126.com)) or to P.Y (ypenghuiamms@hotmail.com) or to J.Y (jane.yu@uc.edu)

**Supplementary information Figure S1**

**Supplementary information, Figure S1**  The level of plasma Angiotensin Ⅱ from RSV-infected human cases at different time-points post infection. AngⅡ levels were detected using radioimmunoassay at the days after onset of RSV symptoms.

**Supplementary information Figure S2**

Figure2 ACE2 deficiency increases the severity of lung injury in mice infected with A2. (a) Kaplan-Meier survival curves were presented. n=10 mice per group. (b) Weight changes in WT or ACE2 KO mice were recorded, n=10 mice per group.(c) Wet to dry weight ratios of the lungs were analyzed after 5 DPI. n=4-6 mice per group.(d) virus load in WT or ACE2 KO mice lung were determined 5 days after RSV A2 infection, n=4-6 mice per group.

**Supplementary information Figure S3**

Figure 3 Recombinant hACE2 reduces the severity of lung injury in Mice infected with A2. B6 mice were injected with Recombinant hACE2 or PBS as a vehicle control 1day prior, as well as 1 day and 3 day after challenged of PBS or live RSV A2 virus. (a) Lung histopathology of PBS and A2 challenged mice treated or untreated with Recombinant hACE2. Scale bar=200μm. The numbers of infiltrating cells are shown for day 5 after infection. n=100 field analyzed for 3 mice for treatment.(b) Wet to dry weight ratios of the lungs were analyzed after 5 DPI. n=4-6 mice per group.(c) virus load in treated or untreated mice lung were determined 5 days after RSV A2 infection, n=4-6 mice per group.

**Supplementary information Figure S4**

Figure 4 ATR2 inhibitor cannot reduce the severity of lung injury or clear up the virus load in Mice infected with A2.(a) B6 mice were injected with ATR2 inhibitor or PBS as a vehicle control 1day prior, as well as 1 day and 3 day after challenged of PBS or live RSV A2 virus. (b) Wet to dry weight ratios of the lungs were analyzed after 5 DPI. n=4-6 mice per group.(c) virus load in treated or untreated mice lung were determined 5 days after RSV A2 infection, n=4-6 mice per group.

**Supplementary information Figure S5**

Figure 5 ATR2 inhibitor cannot reduce the severity of lung injury or clear up the virus load in KO Mice infected with A2.(a) B6 mice were injected with ATR2 inhibitor or PBS as a vehicle control 1day prior, as well as 1 day and 3 day after challenged of PBS or live RSV A2 virus. (b) Wet to dry weight ratios of the lungs were analyzed after 5 DPI. n=4-6 mice per group.(c) virus load in treated or untreated mice lung were determined 5 days after RSV A2 infection, n=4-6 mice per group.

**Supplementary information Figure S6**


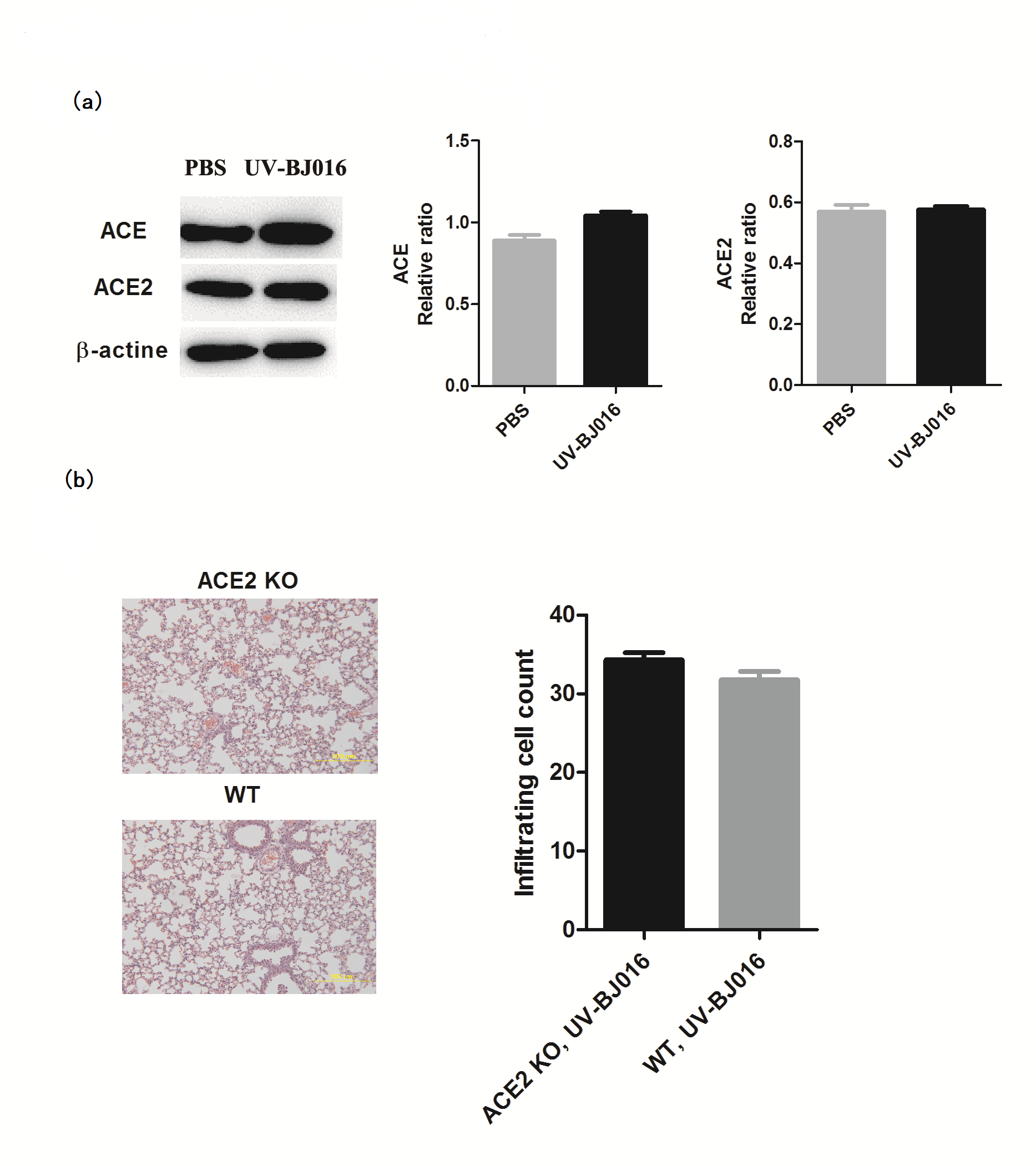


Figure 6. Loss of ACE2 does not affect UV-BJ016 virus infected mice. (a) ACE2 and ACE expression in lung homogenates of PBS and UV-BJ016 infected mice were detected by Western blotting. Mice were sacrificed on day 3 post infection. (b) Lung histopathology of UV-BJ016 infected WT or ACE2 KO mice, n = 4‒6 mice per group. Numbers of infiltrating cells are shown on day 5 after infection. N = 100 fields analyzed.

| Table 1 Detail information of RSV infected patients recruited | | | | | | |
| --- | --- | --- | --- | --- | --- | --- |
| Detail information of infected RSV infected patients recruited | | | | | | |
| Sample | Age | Gendar | Blood-collecting date | Illness onset date | AngⅡ Conc (pg/ml) | Outcome |
| case 1 | 4 month | female | 20130221 | 6 | 711.25 | recovery |
| case 2 | 3 year | male | 20130220 | 8 | 670.75 | recovery |
| case 3 | 3 year | male | 20130209 | 5 | 648 | recovery |
| case 4 | 2 year | female | 20130215 | 3 | 653.75 | recovery |
| case 5 | 2 year | male | 20130219 | 3 | 670.75 | recovery |
| case 6 | 3 month | male | 20130225 | 3 | 659.5 | recovery |
| case 7 | 3 year | male | 20130218 | 7 | 463.5 | recovery |
| case 8 | 4 month | male | 20140102 | 7 | 539.75 | recovery |
| case 9 | 7 month | female | 20140116 | 14 | 53.04 | recovery |
| case 10 | 11 month | male | 20140117 | 11 | 134 | recovery |
| case 11 | 6 month | male | 20140126 | 3 | 253.71 | recovery |
| case 12 | 5 month | male | 20140218 | 14 | 87.27 | recovery |
| case 13 | 7 month | male | 20140220 | 4 | 149.94 | recovery |
| case 14 | 2 month | male | 20140220 | 6 | 521.28 | recovery |
| case 15 | 3 year | female | 20140125 | 7 | 612.00 | recovery |
| case 16 | 3 year | male | 20140126 | 8 | 754.50 | recovery |
| case 17 | 4 month | male | 20140117 | 7 | 491.30 | recovery |
| case 18 | 5 month | male | 20140121 | 8 | 317.10 | recovery |
| case 19 | 2 month | female | 20140126 | 7 | 492.00 | recovery |
| case 20 | 2 year | female | 20140220 | 8 | 567.0 | recovery |
| case 21 | 7 month | male | 20140215 | 8 | 549.4 | recovery |
| case 22 | 3 year | female | 20140216 | 8 | 682.1 | recovery |
| case 23 | 2 year | male | 20140205 | 7 | 669.8 | recovery |
| case 24 | 2 month | female | 20140109 | 1 | 144.13 | recovery |
| case 25 | 1 month | male | 20140207 | 1 | 399.59 | recovery |
| case 26 | 1 month | male | 20140121 | 11 | 228.78 | recovery |
| case 27 | 7 month | male | 20140106 | 10 | 118.13 | recovery |
| case 28 | 9 month | female | 20140221 | 11 | 116.42 | recovery |
| case 29 | 2 month | female | 20140205 | 13 | 252.77 | recovery |
| case 30 | 4 month | female | 20140220 | 13 | 148.35 | recovery |
| case 31 | 3 year | male | 20140205 | 14 | 153.98 | recovery |
| case 32 | 2 month | male | 20140215 | 14 | 149.94 | recovery |
| case 33 | 2 year | male | 20140222 | 8 | 623.75 | recovery |
| case 34 | 3 month | female | 20140115 | 13 | 237.7 | recovery |

| **Table 2 Clinical information for 20 healthy children and** | | | | | | | |
| --- | --- | --- | --- | --- | --- | --- | --- |
| **34 RSV-infected patients enrolled in this study** | | | | | | | |
| **20 healthy children** | | | | | | | |
| Age |  |  | |  | |  |  |
|  | Median(range) | |  | | 4.2 years（3-5 years） | | |
|  | Interquartile range | |  | | 0.75(4-4.75 years) | | |
|  | 3 years |  | |  | | 2 subject (10%) | |
|  | 4 years |  | |  | | 12 subjects (60%) | |
|  | 5 years |  | |  | | 6 subjects (30%) | |
| Male |  |  | |  | | 8 subjects (40%) | |
| Female |  |  | |  | | 12 subjects (60%) | |
| **34 RSV-infected patients** | | | | | | | |
|  | Median(range) | |  | | 1.17years(0-3years) | | |
|  | Interquartile range | |  | | 1.67(0.33-2 years) | | |
|  | <1 years |  | |  | | 22 subjects (64.71%) | |
|  | 2 years |  | |  | | 5 subjects (14.71 %) | |
|  | 3 years |  | |  | | 7 subjects (20.58%) | |
| Male |  |  | |  | | 22 subjects (64.71%) | |
| Female |  |  | |  | | 12 subjects (35.29%) | |
| Clinical outcome | |  | |  | |  | |
|  | Recovery |  | |  | | 34 subjects (100%) | |
